# Bacterial contamination of human skin allografts and antimicrobial resistance: a skin bank problem

**DOI:** 10.1186/s12866-018-1261-1

**Published:** 2018-09-24

**Authors:** Karine Lena Meneghetti, Micaela do Canto Canabarro, Letícia Muner Otton, Thaís dos Santos Hain, Mercedes Passos Geimba, Gertrudes Corção

**Affiliations:** Department of Microbiology, Immunology and Parasitology, Institute of Basic Health Sciences, Universidade Federal do Rio Grande do Sul, Sarmento Leite 500, Porto Alegre, 90050-170 Brazil

**Keywords:** Skin allograft, Bacterial contamination., Antimicrobial resistance., Skin bank., Discards.

## Abstract

**Background:**

Bacterial contamination remains the major problem in skin banks, even after antimicrobial treatment, and results in high rates of tissue discarding. This study aimed to analyze bacterial contamination in 32 human skin allografts from the skin bank of Dr. Roberto Corrêa Chem from the Hospital Complex Santa Casa de Misericórdia de Porto Alegre. These samples were already discarded due to microbial contamination. The identification of the bacteria isolated from skin allografts was performed by matrix assisted laser desorption ionization–time of flight. The antimicrobial susceptibility of the isolates to six different classes of antimicrobials was determined using the disk-diffusion agar method, and the evaluation of the inhibitory potential was determined by the minimal inhibitory concentration (50/90) of antimicrobials already used in the skin bank and those that most isolates were susceptible to.

**Results:**

A total of 21 (65.6%) skin samples were contaminated with Gram-positive bacteria: 1 (4.7%) with *Paenibacillus* sp., 12 (61.9%) with *Bacillus* sp., 6 (28.5%) with *Staphylococcus* sp., and 2 (9.5%) with *Bacillus* sp. and *Staphylococcus* sp. Several resistance profiles, including multiresistance, were found among the isolates. Most of the isolates were susceptible to at least one of the antimicrobials used in the skin bank. All isolates were susceptible to amikacin, gentamicin, and tetracycline, which demonstrated the best inhibitory activities against the isolates and were considered as potential candidates for new antimicrobial treatments.

**Conclusions:**

*Bacillus*, *Paenibacillus*, and *Staphylococcus* were isolated from the skin allografts, thus demonstrating the predominance of Gram-positive bacteria contamination. Other factors not related to the resistance phenotype may also be involved in the persistence of bacterial isolates in the skin allografts after antibiotic treatment. Gentamicin, amikacin, and tetracycline can be considered as an option for a more effective treatment cocktail.

**Electronic supplementary material:**

The online version of this article (10.1186/s12866-018-1261-1) contains supplementary material, which is available to authorized users.

## Background

Skin allografts have been an efficient choice for the treatment of several skin lesions and have mainly been used for the temporary wound coverage of severe extensive burns [1]. The use of homologous skin grafts decreases the risk of infection, wound pain, and the frequency of dressing changes as it acts as a mechanical and biological barrier to reduce the loss of water, proteins, electrolytes, and heat through the wound [2]. The major concern regarding the use of allogeneic skin is the risk of disease transmission by potential pathogens, as skin transplant recipients are often immunologically compromised and are consequently more susceptible to infections, (i.e., patients with severe burns) [1].

Microbial contamination is the main reason for tissue discharge in tissue banks [3]. Unlike other tissues, the skin is colonized by skin-associated microorganisms therefore it cannot be considered sterile at the time of harvesting [4]. In addition to the endogenous microbiota, contamination may also originate from the respiratory tract and bowel, or exogenously from the retrieval staff, mortuary [5], or hospital environment [6]. Although superficial decontamination of the donor’s skin with antiseptics is performed before harvesting, the procedure is not indefectible [4]. Skin banks are therefore responsible for ensuring a safe availability of skin for grafting by performing microbiological screening before and after processing the allogeneic skin.

The skin bank of Dr. Roberto Corrêa Chem from the Hospital Complex Santa Casa de Misericórdia de Porto Alegre was founded in 2005 and was the first skin bank created in Rio Grande do Sul. It was the second skin bank in Brazil, but for a long time it was the only one in effective operation [7]. In the processing of skin allograft, the microbiological screening is at the moment of tissue obtainment. After being harvested, strips of allogenous skins are sampled by passing a sterile swab over their entire length, on both sides, and by collecting a fragment from each tip of the strips, that are then cultured in appropriate media. Only allogeneic skins that do not present microbial growth or growth of acceptable microorganisms (considered non-pathogenic) proceed to the next phase of processing. Skin samples with nonacceptable microorganisms are immediately discarded. Allogeneic skins with acceptable microorganisms are then submitted to a first cycle of treatment with penicillin and streptomycin and, if necessary, a second cycle of treatment with vancomycin. Therefore only skins with negative microbiological cultures after treatment are released for grafting. However, as in other skin banks [3, 8], the skin bank of Dr. Roberto Corrêa Chem faces the problem of discards due to persistent bacterial contamination.

Tissue decontamination is a challenge for tissue banks, and currently there is little consensus about the combination and concentration of antibiotics to be used, which highlights the need for more research on this subject [9, 10]. Most of the published manuscripts only identify the contaminants from skin grafts or test the different antimicrobial cocktails on skin, they do not assess the bacteria’s susceptibility to the antimicrobials used or test these cocktails on nonskin bacteria.

This study aimed to perform a bacteriological analysis by identifying bacteria from human skin allografts and analyzing their antimicrobial susceptibility profile by evaluating the inhibitory potential. The inhibitory potential was calculated from the minimal inhibitory concentration (MIC) (MIC_50_ and MIC_90_) of the antimicrobials already used in the skin bank and those that most isolates were susceptible to. The results should contribute to a better understanding of which factors are contributing to the persistence of bacterial contamination in skin allografts. This initial evaluation of the susceptibility profile should also provide changes in the antimicrobial cocktails used in the treatment of skin allografts.

## Methods

### Ethics

This research was approved by the Research Ethics Committees of the Universidade Federal do Rio Grande do Sul (protocol CAAE 36949514.8.0000.5347) and the Irmandade da Santa Casa de Misericordia de Porto Alegre (protocol CAAE 45100215.1.0000.5335).

### Skin samples

A total of 32 batches of human skin samples procured from cadaveric donors between July 2012 to November 2014, were available from the skin bank of Dr. Roberto Corrêa Chem from the Hospital Complex Santa Casa de Misericórdia de Porto Alegre. The skin allografts were harvested from donors with encephalic death (multi-organ donors) and those who suffered cardiorespiratory arrest. The donors were only accepted after family informed consent for recovery, serology and the use of the skin for research purposes; social and medical history of the donor and physical examination. Briefly, allogeneic skin is procured in aseptic conditions, in operating room with adherence to standards and guidelines. Removal of tissues is carried out until 12 h after the interruption of blood circulation, if the cadaver was kept at room temperature, or up to 24 h if the cadaver was refrigerated at 4 °C ± 2 °C within 06 (six) hours after disruption of blood circulation. The recovery areas are trunk, thighs and for male donors also the legs. Each donor is divided into two batches: batch 1 (tissue harvested from anterior sites of the body) and batch 2 (back sites of the body), being that all processing steps are performed individually for each batch. After the tricotomy of the donor areas, disinfection with chlorhexidine degermant followed by dyeing with alcoholic chlorhexidine are performed. Skin strips 5 × 20 cm thick are harvested with an electric dermatome. A microbiological screening is done by culturing the swab and the tip fragments of the skin strips. Then, skin samples are placed in 50% glycerol to be transported to the skin bank to be performed the next processing procedures according to microbiological results. Microbiological analyzes are performed at all stages of the skin bank processing. If rthe considered nonacceptable microorganisms: aerobic or anaerobic Gram-negative bacilli, Gram-negative cocci, *Clostridium* sp., *Bacillus anthracis, Streptococcus pyogenes* (beta hemolytic); *Staphylococcus aureus*; *Enterococcus* sp. and filamentous fungi or yeasts, the tissue is discarded. In case of contamination by other microorganisms, the skin must undergo antibiotic treatment, first cycle with penicillin (1000 U/mL) and streptomycin (200 mg/mL) and second cycle of vancomycin (50 mg/mL), if the first cycle fails. Skins free from contamination are stored in 85% glycerol and maintained at + 4 ± 2 °C up to two years, being available for grafting.

The 32 skin batches were harvested from 28 donors, 17 corresponding to batch 1 and 15 to batch 2. The donors age ranged from 10 to 72 years (mean 35.56 years), 16 females and 12 males. All batches had already been discarded due to microbial contamination with nonacceptable microorganisms or to the persistence of bacterial contamination, after the first or the second cycle of treatment performed in the skin bank.

### Bacterial culture, isolation, and identification

After a sterile washing step with 0.85% sodium chloride solution in a laminar air flow cabinet, 1 cm^2^ of each skin strip (representing the totality of the sample) was transferred to 80 mL of tryptone soya broth (TSB) incubated at 37 °C for 24 h in aerobic conditions and in 80 mL of thioglycolate broth (TGB) incubated at 37 °C for 48 h in anaerobic conditions. If no turbidity was detected within this period, the samples were maintained for up to 14 days under the conditions described above [8].

For bacterial isolation, an aliquot of the skin culture in TSB and TGB was inoculated in blood agar, mannitol salt agar, MacConkey agar, and eosin methylene blue agar in duplicate. Culture mediums inoculated from TSB were incubated in aerobiose and those inoculated from TGB were incubated in anaerobiosis, both at 37 °C for 24 and 48 h, respectively. Five isolates with equal colony morphology were chosen from the agar plates. All isolated colonies were stored in Broth Heart Brain Infusion (BHI) with 15% glycerol at − 20 °C. For Gram-positive cocci, tube coagulase and DNase tests were performed to differentiate *Staphylococcus aureus* from coagulase-negative staphylococci (CNS).

Isolates were identified using matrix assisted laser desorption ionization–time of flight (MALDI-TOF) (Microflex Biotyper 4.0, Bruker) according to the manufacturer’s specifications. A recent culture colony, or 1 μL of supernatant from the extraction procedure, was directly spotted onto the MALDI plate. This was overlaid with 1 μl of saturated α-cyano-4-hydroxycinnamic acid and allowed to dry. The loaded plate was placed in the instrument for reading. The spectra were analyzed using MALDI Biotyper 4.0 software by standard pattern matching with a default setting. Scores > 2 were considered to indicate species identification, while scores of 1.70–1.99 indicated identification at the genus level. Scores under 1.70 indicated no significant similarity of the spectra with any database entry.

### Antibiotic susceptibility tests

Susceptibility to the antimicrobials (Oxoid™ Disks), the benzylpenicillin penicillin G 10 U (PEN), cefoxitin 30 μg (CFO), ciprofloxacin 5 μg (CIP), amikacin 30 μg (AMI), gentamicin 10 μg (GEN), tetracycline 30 μg (TET), streptomycin 10 μg (EST), and azithromycin 15 μg (AZM) was determined using the disk-diffusion method. Colonies cultured from tryptone soya agar (TSA) were suspended in sterile saline, brought to turbidity 0.5 of the McFarland standard and streaked across Mueller Hinton agar (MHA). After 10–15 min, antimicrobial disks were placed on MHA, incubated at 35 °C for 24 h. Inhibition halos were read following the interpretative criteria diameter zone for staphylococci [11–13], with the exception of EST where the interpretative criteria diameter zone for *Enterococcus* sp. was used [14]. *S. aureus* ATCC 29213 was used as a control.

The MIC of the antimicrobials to which the isolates were all susceptible (AMI, GEN, and TET) and those used in the skin bank (PEN, EST, and vancomycin (VAN)) was assessed by MIC gradient strips (M.I.C.Evaluators™, Oxoid). Colonies were suspended in sterile saline (turbidity equivalent to 0.5 McFarland standard) and streaked across MHA. The strips were placed on MHA after 10–15 min, incubated at 35 °C for 24 h, and read according to the manufacturer’s specifications. To describe the efficacy of the antimicrobials, MIC_50_ (MIC value at which ≥50% of the isolates were inhibited) and MIC_90_ (MIC value at which ≥90% of the isolates were inhibited) values were calculated. Apart from EST, which does not have a defined breakpoint value, all MIC breakpoints were interpreted following CLSI M100-S25 [11] for Gram-positive cocci and CLSI M45-A2 [15] for Gram-positive bacilli.

## Results

### Bacterial isolation and identification

Of the 32 evaluated skin samples, 21 (65.6%) were positive for bacterial culture with Gram-positive bacteria, among which two had previously been submitted to the first cycle of antimicrobial treatment and five to the first and second treatment in the skin bank. Considering the MALDI-TOF score criteria, species of *Bacillus, Staphylococcus*, and *Paenibacillus* were identified. Genera whose species were not identified were designated by sp. or by coagulase test result for Gram-positive cocci, as can be seen in Table 1*.* No Gram-negative bacterium was isolated from the evaluated skin samples. A total of 790 bacterial isolates were obtained, 507 (65%) Gram-positive bacilli and 283 (35%) Gram-positive cocci*.*

**Table 1:** Identification and prevalence of bacteria isolated from human skin grafts

Isolated bacteria	Prevalence in skin samples (*n* = 21)
Gram-positive bacilli	
*Bacillus cereus*	7 (33.3%)
*Bacillus subtilis*	7 (33.3%)
*Bacillus* sp.	7 (33.3%)
*Bacillus vallismortis*	3 (14.2%)
*Bacillus pumilus*	1 (4.7%)
*Bacillus licheniformis*	1 (4.7%)
*Paenibacillus* sp.	1 (4.7%)
Gram-positive cocci	
*Staphylococcus epidermidis*	4 (19%)
*Staphylococcus aureus*	2 (9.5%)
*Staphylococcus capitis*	1 (4.7%)
*Staphylococcus saprophyticus*	1(4.7%)
*Staphylococcus lugdunensis*	1(4.7%)
*Staphylococcus haemolyticus*	1(4.7%)
Coagulase-negative staphylococci	1(4.7%)

### Susceptibility profile to antimicrobials

The antimicrobial susceptibility profile was evaluated in two isolates with equal colony morphology among those isolated from each medium, totaling in 195 Gram-positive bacilli and 124 Gram-positive cocci analyzed. All Gram-positive bacilli were susceptible to CIP, AMI, GEN, and TET, and 92 (48.7%) of these were susceptible to all other antimicrobials tested (Table 2). They presented seven distinct antimicrobial resistance profiles (Table 2). Twenty-eight (14.3%) isolates were resistant to a single antimicrobial, 64 (51.6%) were resistant to two antimicrobials, and 11 (5.6%) were resistant to three antimicrobials. The PEN–CFO resistance profile was the most prevalent, being found in all *Bacillus cereus*. A total of 11 (5.6%) isolates, including *B. cereus* and *Paenibacillus* sp., were resistant to three different classes of antimicrobials and were therefore classified as multidrug resistant (MDR) [16].

**Table 2:** Susceptibility profile of Gram-positive bacilli and Gram-positive cocci isolated from human skin allografts with and without antimicrobial treatment

Gram-Positive Bacilli						Gram-Positive Cocci					
Antimicrobial resistance profile	Number of isolates (*n* = 195)	Isolates	Treatment			Antimicrobial resistance profile	Number of isolates (*n* = 124)	Isolates	Treatment		
			WT	1ST	1ST/2ND				WT	1ST	1ST/2ND
PEN CFO	27 1	*B. cereus* *B. subtilis* *B. licheniformis* *Bacillus* sp. *B. vallismortis*	2 1 9 3 1	— — — —	12 — — — —	PEN CFO AZM	20 1 13	*S. aureus* *S. lugdunensis* CNS *S. haemolyticus* CNS	19 1 — 12 —	— — 1 — 1	— — — — —
PEN–CFO CFO–EST PEN–AZM	59 4 1	*B. cereus* *Paenibacillus* sp. *B. cereus*	28 — —	2 4 —-	29 — 1	PEN–CIP PEN–AZM CIP–AZM	9 1 1	*S. aureus* *S. epidermidis* *S. epidermidis* *S. epidermidis*	5 4 1 1	— — — —	— — — —
PEN–CFO–EST PEN–CFO–AZM	10 1	*B. cereus* *Paenibacillus* sp. *B. cereus*	— — —	— 2 —	8 — 1	CFO–CIP–AZM	6	*S. epidermidis* *S. saprophyticus*	5 1	— —	— —
						PEN–CFO–CIP–AZM	54	*S. epidermidis* *S. capitis* *S. saprophyticus* *S. aureus*	35 17 1 1	— — — —	— — — —
Susceptible to all ATM	92	*Bacillus* sp. *B. subtilis* *B. vallismortis* *B. pumilus* *B. licheniformis* *B. cereus*	49 25 4 2 1 1	2 — — — — —	4 4 — — — —	Susceptible to all ATM	19	*S. haemolyticus* *S. lugdunensis*	4 15	— —	— —

1ST, isolates obtained from skin samples previously submitted to the first cycle of treatment with penicillin and streptomycin in the skin bank; *2ND*, isolates obtained from skin samples previously submitted to the second cycle of treatment with vancomycin; *ATM*, antimicrobials; *AZM*, azithromycin; *CFO*, cefoxitin; *CIP,* ciprofloxacin; *CNS*, coagulase-negative staphylococci; *EST*, streptomycin; *PEN*, penicillin; *WT*, without treatment

Among the 102 (52%) PEN- and EST-resistant bacilli isolates, 59 were isolated from skin samples submitted to the first and second treatment cycles with these antimicrobials and 49 (83%) were susceptible to at least one of them. Another 10 bacilli from treated skin samples were susceptible to both of them (Table 2).

All of the 124 Gram-positive cocci analyzed were susceptible to AMI, GEN, and TET and 19 (15.3%) were susceptible to all other antimicrobials tested (Table 2). Eight antimicrobial resistance profiles were observed, of which PEN–CFO–CIP–AZM was the most prevalent being observed in 54 (43.5%) Gram-positive cocci. A total of 60 (48.3%) isolates were classified as MDR, being resistant to three or more different classes of antimicrobials, including *S. aureus, S. saprophyticus, S. epidermidis*, and *S. capitis*. In addition, 61 (49.1%) staphylococci isolates were oxacillin-resistant based on CFO results predicting methicillin resistance (Table 2).

A total of 84 (67.7%) Gram-positive cocci were resistant to PEN and no coccus isolates showed resistance to EST. The two CNS isolated from skin samples submitted to the first treatment in the skin bank were susceptible to both PEN and EST (Table 2).

### Antimicrobial inhibitory potential

The inhibitory potential of the antimicrobials to which all isolates were susceptible by disk-diffusion method (AMI, GEN, and TET) and those used in the skin bank (PEN, EST, and VAN) were evaluated against the isolated bacteria to assess their efficiency (Table 3). For this purpose, 103 Gram-positive cocci and 118 Gram-positive bacilli with different susceptibility profiles were chosen and analyzed using MIC gradient strips.

**Table 3:** MIC_50_ and MIC_90_ values of penicillin, streptomycin, vancomycin, amikacin, gentamicin, and tetracycline for Gram-positive bacilli and cocci

Isolates	μg/mL	PEN	EST	VAN	AMI	GEN	TET
Gram-positive bacilli (*n* = 118)	MIC_50_	1	2	2	0.5	0.5	0.5
	MIC_90_	> 32	32	8	1	2	8
Gram-positive cocci (*n* = 103)	MIC_50_	8	4	2	2	0.5	1
	MIC_90_	> 32	8	4	4	2	2
Gram-positive bacilli and cocci (*n* = 221)	MIC_50_	8	4	2	0.5	0.5	1
	MIC_90_	> 32	16	8	2	2	4

*AMI*, amikacin; *EST*, streptomycin; *GEN*, gentamicin; *MIC*_*50*_, MIC value at which ≥50% of the isolates were inhibited; *MIC*_*90*,_ MIC value at which ≥90% of the isolates were inhibited *PEN*, penicillin; *TET*, tetracycline; *VAN*, vancomycin

For Gram-positive bacilli PEN MIC_50_ was 1 μg/mL and for Gram-positive cocci PEN MIC_50_ was 8 μg/mL, and both presented PEN MIC_90_ values of > 32 μg/mL. EST MIC_50_ was 2 μg/mL and EST MIC_90_ was 32 μg/mL for Gram-positive bacilli while for Gram-positive cocci EST MIC_50_ and MIC_90_ was 4 μg/mL and 8 μg/mL, respectively. VAN MIC_50_ was 2 μg/mL for Gram-positive bacilli and cocci, and VAN MIC_90_ was 8 μg/mL for Gram-positive bacilli and 2 μg/mL for Gram-positive cocci. AMI MIC_50_ of 0.5 μg/mL and MIC_90_ of 1 μg/mL was showed by Gram-positive bacilli while for Gram-positive cocci was AMI MIC_50_ of 2 μg/mL and MIC_90_ of 4 μg/mL. GEN MIC_50_ of 0.5 μg/mL and MIC_90_ of 2 μg/mL was observed for both Gram-positive bacilli and cocci. TET MIC_50_ was 0.5 μg/mL and MIC_90_ was 8 μg/mL for Gram-positive bacilli. For Gram-positive cocci TET MIC_50_ was 1 μg/mL and MIC_90_ was 2 μg/mL (Table 3).

Considering all the 221 Gram-positive bacilli and cocci avaluated, for elimination of 90% of them was observed MIC values of > 32 μg/mL for PEN, 16 μg/mL for EST, 8 μg/mL for VAN, 2 μg/mL for AMI and GEN, and 4 μg/mL for TET. Therefore,

AMI and GEN presented the highest inhibitory activity, followed by TET. Both AMI, GEN and TET presented MIC_90_ values less than the susceptibility breakpoints established by CLSI M45-A2 [15] and CLSI M100-S25 [11], respectively, for *Bacillus* sp. and *Staphylococcus sp*., with exception TET MIC_90_ that was equal to the intermediate resistance breakpoint for *Bacillus* sp., indicating the absence of resistance to these antimicrobials. Meanwhile, PEN, EST, and VAN were the least active, requiring higher concentrations to inhibit the growth of the isolates. PEN MIC_90_ was 128-fold higher than the breakpoint of resistance (≥0.25 μg/mL, CLSI M45-A2 [15], CLSI M100-S25 [11]) for *Bacillus* sp. and *Staphylococcus* sp., demonstrating high PEN resistance among the isolates. VAN MIC_90_ was two-fold higher than the breakpoint of susceptibility (≤4 μg/mL, CLSI M45-A2 [15]) for *Bacillus* sp., suggesting that some bacilli isolates were nonsuceptible to VAN. Moreover VAN MIC_90_ presented equal value of the breakpoint of intermediate resistance for *Staphylococcus* sp. (Table 3).

Regarding the bacteria isolated from skin samples previously submitted to the first treatment with PEN in the skin bank, individual MIC analysis revealed that 43 (93.4%) of the 46 Gram-positive bacilli were PEN resistant, presenting PEN MIC values between 0.25 and ≥ 32 μg/mL. Three of the isolates (6.5%) were susceptible to PEN with MIC values between 0.03 and 0.06 μg/mL. Two of the Gram-positive cocci were susceptible to PEN with MIC values of between 0,06 and 0,12 μg/mL (Additional file 1).

Of the 39 Gram-positive bacilli submitted to the second cycle of treatment with VAN, 18 (46.1%) were shown to be susceptible and presented MIC values between 0.25 and 2 μg/mL. However, 21 (53.8%) Gram-positive bacilli were resistant to VAN, showing MIC values between 8 and 16 μg/mL (Additional file 1). No skin sample, which was submitted to the second cycle of treatment with VAN, presented Gram-positive cocci.

## Discussion

The primary aim of this study was to analyze the bacterial contaminants of allogeneic skin samples from the skin bank of Dr. Roberto Corrêa Chem from the Hospital Complex Santa Casa de Misericórdia de Porto Alegre. *Bacillus* sp., *Staphylococcus* sp., and *Paenibacillus* sp. were identified. The most prevalent Gram-positive bacilli was *B. cereus* and the most prevalent Gram-positive cocci was *S. epidermidis*.

Another Brazilian skin bank has also reported skin contamination by Gram-positive bacteria, including CNS (42%), Gram-positive bacilli (not *Clostridium*) (18%), coagulase-positive staphylococci (10%), and *Enterococcus* sp. (7%) [17]. Lindford et al. [18] found that *Staphylococcus* and *Bacillus* were the most prevalent bacteria in skin allografts*.* Other authors [1, 3, 8] have identified CNS, especially *S. epidermidis*, as the most common bacteria in skin allografts*.* However, other studies have also detected Gram-negative bacteria such as *Escherichia coli, Klebsiella pneumoniae, Pseudomonas aeruginosa, Proteus* sp.*, Acinetobacter baumannii*, and *Enterobacter cloacae* [1, 3, 8, 18]. The absence of Gram-negative bacilli in the present work may be due to the fact that the analyzed skin samples were immersed in 85% glycerol for long periods of time, the inhibitory action of which is more potent in Gram-negative bacteria. The higher susceptibility of Gram-negative bacteria to high glycerol concentrations is due to the thin layer of peptidoglycan, which constitutes only 10% of the cell wall. Conversely, the peptidoglycan content in Gram-positive bacteria corresponds to 90% of the cell wall and is therefore more resistant to osmotic lysis [19]. Studies have also shown that glycerol is unable to sterilize skin as it cannot eliminate bacterial spores [4, 19].

*Bacillus* sp. and *Paenibacillus* sp. are noteworthy as they form spores that are resistant to heat, cold, and common disinfectants, which allows them to survive on surfaces for prolonged periods [20]; *Bacillus* sp. have been found even on the skin surface of healthy individuals [21]. There are few methods that reduce skin spore levels in a clinical setting, mainly in hospitalized patients [22]. *Bacillus* sp. and *Paenibacillus* sp. have been isolated in these individuals [23]. In addition to forming spores, these bacteria seem to be sufficiently equipped with virulence properties that allow them to behave as pathogens and opportunistic pathogens in humans [23].

Among the *Staphylococcus* species, *S. epidermidis, S. haemolyticus, S. capitis*, and *S. lugdunensis* can be considered to be an integral part of the normal skin microbiota. However, although less virulent, these coagulase-negative species may cause infections [24]. They are particularly associated with infections caused by the use of implanted devices and they have a high capacity for biofilm formation, which shows significant resistance to antibiotics [25].

Several species detected in this study (i.e., *Paenibacillus* sp. [26], *B. cereus* [27], *B. subtilis* [28], *B. pumilus* [29], *B. licheniformis* [30], *S. aureus* [31], *S. epidermidis* [24], and *S. capitis* [32]) have already been shown to directly cause infections in the skin, open-wounds, or the blood (via skin lesions). With the exception of *S. aureus* and *B. cereus,* most of the bacteria isolated in this study are considered to be of low pathogenicity [3], including normal human skin microbiota and common environmental organisms. However, they should not be underestimated as they are isolated from skin grafts that are usually transplanted into immunocompromised individuals with a high risk of acquiring a serious opportunistic infection [18].

Few articles discuss about susceptible profiles to antimicrobials of bacteria isolated from skin allografts. This is an important tool to guide the choice of an appropriate antibiotic treatment, especially if the applied treatments are not completely efficient. Among the 319 isolates that had their susceptible profiles determined, 71 (22.2%) were multidrug resistant. This is not the first time that MDR bacteria have been reported in tissue bank samples [33]. Pianigiani et al. [34] found MDR Staphylococci isolated from skin allografts, that called for treatment with vancomycin on the basis of antibiograms.

Regarding the MIC values, it was observed high levels of resistance to PEN in a portion of the population of the isolates evaluated, in addition to nonsuceptible isolates and intermediate resistant to VAN. In view of this, the resistance to the antimicrobials applied in the decontamination treatment of the skin allografts might be contributing to its inefficiency.

The inhibitory potential of the antimicrobials used in the skin bank showed to be less effective, requiring high concentrations to kill the isolates evaluated. Considering all the 221 isolates of bacilli and cocci, PEN showed MIC90 values higher than their breakpoint of resistance. VAN MIC90 presented equal value of the breakpoint of intermediate resistance for *Staphylococcus* sp. and nonsuceptible breakpoint for *Bacillus* sp. (including *Paenibacillus* sp.). Streptomycin did not have reference values, but among the antimicrobials used in the skin bank, presented the second highest value of MIC90 (16 μg/mL). Interestingly the MIC values of these three antimicrobials were much lower than the concentrations used in the skin bank, which concentrations might have eliminated at least the bacteria susceptible to them. It is possible that such concentrations were quite ineffective against susceptible bacteria due to the ‘Eagle effect’, where antibiotics, particularly β-lactams, exhibits reduced potential killing at high drug concentrations [9, 35]. AMI, GEN and TET presented MIC90 values less than the susceptibility breakpoints established for the isolates, being much more effective at lower concentrations than the antimicrobial concentrations already used in the skin bank.

These findings highlights the need to find different alternatives of antimicrobial treatments and a monitoring of resistance within the tissue banks, since broad spectrum antimicrobials are used and can select resistant bacteria, making it even more difficult to eliminate contamination from tissue allografts. In addition, the concentration of antimicrobials should also be better studied, so that an ideal value is reached, which eliminates the greater amount of bacteria found in the skin allografts, without selecting resistant strains [36] nor a high concentration that loses its effect [35].

Little information is available regarding the treatment of infections caused by *Bacillus* or *Paenibacillus* strains [23]. *B. cereus* are described as potent producers of broad-spectrum β-lactamases, which affect penicillins and cephalosporins [15]. This may explain their high levels of resistance to PEN, CAZ, and CFO. *B. cereus* is known to be susceptible to aminoglycosides, chloramphenicol, clindamycin, erythromycin, TET, and VAN [37], therefore of the antimicrobials tested in this study, AMI, GEN, and TET may be efficient at eliminating vegetative forms of *Bacillus* species.

Methicillin-resistant staphylococci (MRS) is considered to be resistant to all currently-available β-lactam antimicrobial agents except for cephalosporins, which have anti-methicillin-resistant *Staphylococcus aureus* (anti-MRSA) activity [11]. Thus the elaboration of a new treatment cocktail should take this into consideration.

Previous studies have already shown that antibiotic resistance has increased in skin isolates, which may reflect the indiscriminate use of antibiotics over the last few years [21]. Cosmetic products containing antibiotics and disinfectants may also be associated with this resistance [38]. Hospital environments such as intensive care are populated by antibiotic-resistant species [34] and considering that some skin donors might have previously undergone hospitalization, the characteristics of their microbiota may have been modified by the bacteria present in this environment, both in terms of their composition [3, 6] as their resistance to antimicrobials [39].

Although some isolates presented a MDR profile, most of the isolates submitted to the first treatment with PEN and EST were susceptible to one or both of these antimicrobials. This suggests that other factors that are unrelated to antimicrobial resistance may be contributing to the persistence of these bacteria in skin allografts. The inefficiency of EST to eliminate bacteria may be due to the fact that aminoglycosides are indicated in combination with other active agents, (i.e., β-lactam) [11]. This may explain why EST alone was not able to eliminate PEN-resistant staphylococci isolates.

Pirnay et al. [8] tested a cocktail (TM1) containing penicillin, EST, and amphotericin B and observed that it failed to decontaminate 47.4% of skin grafts tested. Of the seven skin samples that were treated with PEN and EST in this study, the contamination was also shown to persisted. Pirnay et al. also tested a second cocktail (TM2) composed of GEN sulfate, imipenem/cilastin, polymyxin B sulfate, VAN HCl, and nystatin and found that it was also unable to decontaminate the skin samples. Disk-diffusion analysis demonstrated that bacteria from samples treated with TM2 were susceptible to at least one of the antibiotics present in the cocktail, as has been observed in this study. Gaucher el al. [3] also reported the persistence of VAN-susceptible bacteria even after skin allografts were treated with a cocktail containing high concentration of this antimicrobial.

Some reasons may cause antimicrobial skin decontamination to be inefficient even if the bacteria are susceptible to them. For example, spore-forming bacteria can remain dormant for long periods and are extremely resistant to the action of antimicrobials [40]. Bacteria may also remain within pores or hair follicles and thus evade the action of antimicrobials that cannot reach such sites [41]. Some bacteria may also form biofilms on the skin [42]. Some studies have already reported biofilm formation in other types of grafts, such as bone [43] and prosthetic vascular grafts [44].

The organization of bacterial cells into a biofilm may cause them to be more tolerant to the effects of antimicrobial agents; even bacteria that do not have a genetic basis for antimicrobial resistance may reduce their susceptibility when they form biofilms [45]. It is important to highlight that most of the antimicrobials used in the clinic only target cells in the planktonic state. Furthermore, some antimicrobial agents may stimulate the production of biofilms by certain microorganisms [46]. For the treatment of allogeneic skin grafts it may therefore be necessary to incorporate not only antibiotics but also other compounds that interfere with other functions of bacterial cells (i.e., biofilm destabilization or spore elimination).

In this study, all Gram-positive cocci and bacilli were susceptible to AMI, GEN, and TET. Therefore one or more of these antimicrobials could be included into a skin treatment cocktail. Adjustments in the concentrations of antimicrobials already used in the skin bank, or combinations of antimicrobials of different classes, may be more efficient in the process of skin allograft decontamination. Some studies have already tested GEN and AMI in different cocktails for the treatment of skin allografts [6, 47]; however, TET has not yet been tested. This should encourage future studies to combine the antimicrobials with the best inhibitory potential.

## Conclusions

This is the first article to describe a microbiological screening and antimicrobial susceptible profile for bacteria isolated from human skin allografts in a Brazilian skin bank. *Bacillus*, *Paenibacillus*, and *Staphylococcus* were isolated thus demonstrating the predominance of Gram-positive bacteria contamination. Although several isolates presented multiresistance profiles, the persistence of the contamination in the majority of samples was not related to the antimicrobial resistance phenotype. This suggests that other factors may be responsible for the persistence of these bacteria in allogeneic skin. Biofilm formation is one such factor and should be studied further, especially considering that its presence in skin allografts could have a direct influence on the treatment method.

All isolates were susceptible to GEN, AMI, and TET and these antimicrobials showed the best inhibitory potential. Therefore these could be considered as an option for a more effective treatment cocktail.

It is important to share the results of this study to compare and discuss methodologies and findings among different tissue banks. This will help to reduce the rate of discards due to microbial contamination, increase the supply of suitable allogeneic skin, and guarantee greater safety to skin allografts recipients.

## Additional file

Additional file 1: **Table S1.** Susceptibility profile and antimicrobial MIC values of Gram-positive bacilli isolated from human skin allografts with and without antimicrobial treatment. **Table S2.** Susceptibility profile and antimicrobial MIC values of Gram-positive cocci isolated from human skin allografts with and without antimicrobial treatment. (PDF 478 kb)

## Abbreviations

AMI: Amikacin
ATM: Antimicrobials
AZM: Azithromycin
BHI: Broth heart brain infusion
CFO: Cefoxitin
CIP: Ciprofloxacin
CNS: Coagulase-negative staphylococci
EST: Streptomycin
GEN: Gentamicin
MALDI-TOF: Matrix assisted laser desorption ionization–time of flight
MDR: Multidrug resistant
MHA: Mueller Hinton agar
MIC: Minimal inhibitory concentration
PEN: Penicillin
TET: Tetracycline
TGB: Thioglycolate broth
TSA: Tryptone soya agar
TSB: Tryptone soya broth
VAN: Vancomycin
WT: Without treatment
