# Supplementary material for: Bacterial contamination of human skin allografts and antimicrobial resistance: a skin bank problem
Source: BMC Microbiol. 2018 Sep 24;18:121. doi: 10.1186/s12866-018-1261-1 (PMC6154792; doi:10.1186/s12866-018-1261-1)
Supplement: Supplementary file 1 — Table S1. Susceptibility profile and antimicrobial MIC values of Gram-positive bacilli isolated from human skin allografts with and without antimicrobial treatment. Table S2. Susceptibility profile and antimicrobial MIC values of Gram-positive cocci isolated from human skin allografts with and without antimicrobial treatment. (PDF 478 kb) [file 12866_2018_1261_MOESM1_ESM.pdf]

Table S1. Susceptibility profile and antimicrobial MIC values of Gram-positive bacilli isolated from human skin allografts with and without antimicrobial treatment.

| Isolates | Treatment | | | Antimicrobial resistance profile | MIC values (µg/mL) | | | | | |
|---|---|---|---|---|---|---|---|---|---|---|
| | WT | 1ST | 1ST/ 2ND | | PEN | EST | VAN | AMI | GEN | TET |
| *Bacillus cereus* | | X | X | PEN | >32 | 2 | 4 | 1 | 1 | 0,5 |
| *Bacillus cereus* | | X | X | PEN | >32 | 4 | 8 | 0,5 | 0,5 | 0,25 |
| *Bacillus cereus* | | X | X | PEN | >32 | 2 | 8 | 1 | 1 | 0,5 |
| *Bacillus cereus* | | X | X | PEN | >32 | 2 | 4 | 1 | 1 | 1 |
| *Bacillus cereus* | | X | X | PEN | >32 | 4 | 4 | 0,5 | 1 | 0,5 |
| *Bacillus cereus* | | X | X | PEN | >32 | 4 | 8 | 0,5 | 1 | 1 |
| *Bacillus cereus* | | X | X | PEN | >32 | 2 | 4 | 0,5 | 1 | 0,5 |
| *Bacillus cereus* | | X | X | PEN | >32 | 8 | 8 | 0,5 | 0,25 | 0,25 |
| *Bacillus cereus* | | X | X | PEN | >32 | 16 | 8 | 2 | 1 | 0,25 |
| *Bacillus* sp. | X | | | PEN | 0,5 | 1 | 0,5 | 0,25 | 0,12 | 0,5 |
| *Bacillus* sp. | X | | | PEN | 0,03 | 2 | 0,25 | 0,25 | 0,12 | 0,25 |
| *Bacillus* sp. | X | | | PEN | 0.25 | 2 | 0,25 | 0,25 | 0,12 | 0,25 |
| *Bacillus subtilis* | X | | | PEN | 0.03 | 2 | 0,5 | 0,12 | <0,06 | 0,03 |
| *Bacillus licheniformis* | X | | | PEN | >32 | 4 | 1 | 1 | 0,25 | 0,12 |
| *Bacillus licheniformis* | X | | | PEN | >32 | 4 | 2 | 1 | 0,5 | 0,5 |
| *Bacillus licheniformis* | X | | | PEN | >32 | 8 | 0,5 | 1 | 0,5 | 0,25 |
| *Bacillus vallismortis* | X | | | PEN | 0,5 | 1 | 1 | 0,5 | 0,5 | 1 |
| *Bacillus licheniformis* | X | | | PEN | >32 | 16 | 1 | 1 | 0,5 | 1 |
| *Bacillus cereus* | X | | | PEN | >32 | 2 | 4 | 0,5 | 1 | 8 |
| *Bacillus cereus* | X | | | PEN | 4 | 2 | 2 | 0,5 | 1 | 2 |
| *Bacillus vallismortis* | X | | | CFO | 1 | 1 | 2 | 0,25 | 0,12 | 0,25 |

1ST, isolates obtained from skin samples previously submitted to the first cycle of treatment with penicillin and streptomycin in the skin bank; *2ND* ,isolates obtained from skin samples previously submitted to the second cycle of treatment with vancomycin; *AMI*, amikacin; *ATM*, antimicrobials; *AZM*, azithromycin; *CFO*, cefoxitin; *CIP*, ciprofloxacin; *CNS*, coagulase-negative staphylococci; *EST*, streptomycin; *GEN*, gentamicin*; MIC*, minimal inhibitory concentration; *PEN*, penicillin; *TET*, tetracycline; *VAN*, vancomycin*; WT*, without treatment.

Table S1. Continued.

| Isolates | Treatment | | | Antimicrobial resistance profile | MIC values (µg/mL) | | | | | |
|---|---|---|---|---|---|---|---|---|---|---|
| | WT | 1ST | 1ST/ 2ND | | PEN | EST | VAN | AMI | GEN | TET |
| *Bacillus cereus* | | X | X | PEN-CFO | >32 | 4 | 16 | 1 | 2 | 1 |
| *Bacillus cereus* | | X | X | PEN-CFO | >32 | 2 | 16 | 2 | 2 | 1 |
| *Bacillus cereus* | | X | X | PEN-CFO | >32 | 4 | 16 | 2 | 4 | 1 |
| *Bacillus cereus* | | X | X | PEN-CFO | >32 | 4 | 8 | 0,5 | 0,5 | 0,5 |
| *Bacillus cereus* | | X | X | PEN-CFO | >32 | 2 | 4 | 0,5 | 0,5 | 1 |
| *Bacillus cereus* | | X | X | PEN-CFO | >32 | 4 | 4 | 0,5 | 1 | 0,5 |
| *Bacillus cereus* | | X | X | PEN-CFO | >32 | 2 | 4 | 0,5 | 0,5 | 0,25 |
| *Bacillus cereus* | | X | X | PEN-CFO | >32 | 4 | 2 | 1 | 2 | 2 |
| *Bacillus cereus* | | X | X | PEN-CFO | >32 | 2 | 2 | 0,5 | 1 | 2 |
| *Bacillus cereus* | | X | X | PEN-CFO | >32 | 4 | 16 | 1 | 1 | 0,5 |
| *Bacillus cereus* | | X | X | PEN-CFO | >32 | 1 | 8 | 2 | 1 | 0,5 |
| *Bacillus cereus* | | X | X | PEN-CFO | >32 | 4 | 16 | 0,5 | 0,5 | 0,25 |
| *Bacillus cereus* | | X | X | PEN-CFO | 16 | 1 | 8 | 0,5 | 0,5 | 0,5 |
| *Bacillus cereus* | | X | X | PEN-CFO | >32 | 2 | 4 | 0,5 | 0,5 | 1 |
| *Bacillus cereus* | | X | X | PEN-CFO | >32 | 2 | 8 | 1 | 1 | 0,5 |
| *Bacillus cereus* | | X | X | PEN-CFO | >32 | 4 | 8 | 0,5 | 0,5 | 0,5 |
| *Bacillus cereus* | | X | X | PEN-CFO | >32 | 2 | 8 | 0,5 | 0,25 | 0,25 |
| *Bacillus cereus* | | X | X | PEN-CFO | >32 | 16 | 4 | 0,5 | 0,5 | 0,5 |
| *Bacillus cereus* | | X | | PEN-CFO | >32 | 2 | >256 | 0,5 | 0,5 | 4 |
| *Bacillus cereus* | X | | | PEN-CFO | >32 | 4 | 8 | 1 | 2 | 1 |
| *Bacillus cereus* | X | | | PEN-CFO | >32 | 2 | 4 | 1 | 1 | 16 |

1ST, isolates obtained from skin samples previously submitted to the first cycle of treatment with penicillin and streptomycin in the skin bank; *2ND* ,isolates obtained from skin samples previously submitted to the second cycle of treatment with vancomycin; *AMI*, amikacin; *ATM*, antimicrobials; *AZM*, azithromycin; *CFO*, cefoxitin; *CIP*, ciprofloxacin; *CNS*, coagulase-negative staphylococci; *EST*, streptomycin; *GEN*, gentamicin; *MIC*, minimal inhibitory concentration; *PEN*, penicillin; *TET*, tetracycline; *VAN*, vancomycin; *WT*, without treatment.

| Table S1. Continued. | | | | | | | | | | |
|---|---|---|---|---|---|---|---|---|---|---|
| **Isolates** | **Treatment** | | | **Antimicrobial resistance profile** | **MIC values (µg/mL)** | | | | | |
| | **WT** | **1ST** | **1ST/ 2ND** | | **PEN** | **EST** | **VAN** | **AMI** | **GEN** | **TET** |
| *Bacillus cereus* | X | | | PEN-CFO | >32 | 2 | 4 | 1 | 2 | 16 |
| *Bacillus cereus* | X | | | PEN-CFO | >32 | 2 | 8 | 2 | 2 | 16 |
| *Bacillus cereus* | X | | | PEN-CFO | >32 | 4 | 4 | 1 | 1 | 16 |
| *Bacillus cereus* | X | | | PEN-CFO | >32 | 2 | 4 | 1 | 0,25 | 8 |
| *Bacillus cereus* | X | | | PEN-CFO | >32 | 16 | 2 | 0,25 | 0,25 | 2 |
| *Bacillus cereus* | X | | | PEN-CFO | >32 | 4 | 4 | 0,5 | 1 | 4 |
| *Bacillus cereus* | X | | | PEN-CFO | >32 | 4 | 2 | 1 | 1 | 4 |
| *Bacillus cereus* | X | | | PEN-CFO | 16 | 1 | 2 | 1 | 1 | 2 |
| *Bacillus cereus* | X | | | PEN-CFO | 4 | 1 | 4 | 0,5 | 2 | 2 |
| *Bacillus cereus* | X | | | PEN-CFO | >32 | 4 | 2 | 0,5 | 1 | 4 |
| *Bacillus cereus* | X | | | PEN-CFO | >32 | 2 | 2 | 0,5 | 1 | 4 |
| *Bacillus cereus* | X | | | PEN-CFO | >32 | 1 | 2 | 1 | 1 | 2 |
| *Paenibacillus* sp. | | X | | CFO-EST | 1 | >512 | 2 | 0,12 | 0,12 | 0,12 |
| *Paenibacillus* sp. | | X | | CFO-EST | 1 | >512 | 2 | 1 | 1 | 0,25 |
| *Paenibacillus* sp. | | X | | CFO-EST | 0,5 | >512 | 1 | 0,12 | <0,06 | <0,015 |
| *Bacillus cereus* | | X | X | PEN-AZM | >32 | 4 | 8 | 0,5 | 1 | 0,25 |
| *Bacillus cereus* | | X | X | PEN-CFO-EST | 8 | 128 | 4 | 1 | 4 | 4 |
| *Bacillus cereus* | | X | X | PEN-CFO-EST | 16 | 128 | 4 | 0,5 | 1 | 8 |
| *Bacillus cereus* | | X | X | PEN-CFO-EST | 0,06 | 128 | 0,5 | 0,5 | 2 | 0,5 |
| *Bacillus cereus* | | X | X | PEN-CFO-EST | >32 | 128 | 8 | 0,5 | 0,5 | 2 |
| *Bacillus cereus* | | X | X | PEN-CFO-EST | >32 | 128 | 8 | 1 | 1 | 1 |

1ST, isolates obtained from skin samples previously submitted to the first cycle of treatment with penicillin and streptomycin in the skin bank; *2ND* ,isolates obtained from skin samples previously submitted to the second cycle of treatment with vancomycin; *AMI*, amikacin; *ATM*, antimicrobials; *AZM*, azithromycin; *CFO*, cefoxitin; *CIP,* ciprofloxacin; *CNS*, coagulase-negative staphylococci; *EST*, streptomycin; *GEN*, gentamicin*; MIC*, minimal inhibitory concentration; *PEN*, penicillin; *TET*, tetracycline; *VAN*, vancomycin*; WT*, without treatment.

Table S1. Continued.

| Isolates | Treatment | | | Antimicrobial resistance profile | MIC values (µg/mL) | | | | | |
|---|---|---|---|---|---|---|---|---|---|---|
| | **WT** | **1ST** | **1ST/ 2ND** | | **PEN** | **EST** | **VAN** | **AMI** | **GEN** | **TET** |
| *Bacillus cereus* | | X | X | PEN-CFO-EST | >32 | 256 | 8 | 2 | 4 | 1 |
| *Paenibacillus* sp. | | X | | PEN-CFO-EST | 1 | >512 | 1 | 0,12 | <0,06 | 0,015 |
| *Paenibacillus* sp. | | X | | PEN-CFO-EST | 1 | >512 | 1 | 0,25 | 0,06 | 0,03 |
| *Bacillus cereus* | | X | X | PEN-CFO-AZM | >32 | 64 | 8 | 0,5 | 0,5 | 0,5 |
| *Bacillus* sp. | | X | X | Susceptible to all ATM | 0,25 | 1 | 1 | 0,25 | 0,5 | 0,25 |
| *Bacillus subtilis* | | X | X | Susceptible to all ATM | 0,03 | 2 | 2 | 0,25 | 0,12 | 0,12 |
| *Bacillus subtilis* | | X | X | Susceptible to all ATM | 1 | 1 | 0,5 | 0,25 | 0,12 | 0,25 |
| *Bacillus* sp. | | X | X | Susceptible to all ATM | 4 | 1 | 2 | 0,25 | 0,06 | 2 |
| *Bacillus* sp. | | X | | Susceptible to all ATM | 0,03 | 4 | 1 | 0,12 | 0,5 | 2 |
| *Bacillus* sp. | X | | | Susceptible to all ATM | 0,03 | 1 | 0,5 | 0,25 | 0,25 | 8 |
| *Bacillus* sp. | X | | | Susceptible to all ATM | 0,5 | 4 | 0,25 | 0,5 | 1 | 8 |
| *Bacillus* sp. | X | | | Susceptible to all ATM | 0,12 | 2 | 1 | 0,5 | 0,25 | 8 |
| *Bacillus* sp. | X | | | Susceptible to all ATM | 0,25 | 1 | 64 | 1 | 1 | 8 |
| *Bacillus* sp. | X | | | Susceptible to all ATM | 0,25 | 2 | 1 | 1 | 0,5 | 16 |
| *Bacillus* sp. | X | | | Susceptible to all ATM | 0,12 | 1 | 2 | 0,5 | 0,5 | 16 |
| *Bacillus* sp. | X | | | Susceptible to all ATM | 0,12 | 1 | 2 | 0,25 | 0,5 | 8 |
| *Bacillus* sp. | X | | | Susceptible to all ATM | 0,5 | 4 | 1 | 0,25 | 1 | 8 |
| *Bacillus* sp. | X | | | Susceptible to all ATM | 0,015 | 4 | 0,5 | 0,25 | 0,25 | 2 |
| *Bacillus* sp. | X | | | Susceptible to all ATM | 0,25 | 1 | 0,25 | 0,25 | 0,12 | 0,25 |
| *Bacillus* sp. | X | | | Susceptible to all ATM | 1 | 1 | 1 | 0,5 | 0,12 | 0,25 |
| *Bacillus* sp. | X | | | Susceptible to all ATM | 0.015 | 1 | 0,5 | 0,12 | 0,5 | 0,12 |

1ST, isolates obtained from skin samples previously submitted to the first cycle of treatment with penicillin and streptomycin in the skin bank; *2ND* ,isolates obtained from skin samples previously submitted to the second cycle of treatment with vancomycin; *AMI*, amikacin; *ATM*, antimicrobials; *AZM*, azithromycin; *CFO*, cefoxitin; *CIP*, ciprofloxacin; *CNS*, coagulase-negative staphylococci; *EST*, streptomycin; *GEN*, gentamicin; *MIC*, minimal inhibitory concentration; *PEN*, penicillin; *TET*, tetracycline; *VAN*, vancomycin; *WT*, without treatment.

Table S1. Continued.

| Isolates | Treatment | | | Antimicrobial resistance profile | MIC values (µg/mL) | | | | | |
|---|---|---|---|---|---|---|---|---|---|---|
| | WT | 1ST | 1ST/ 2ND | | PEN | EST | VAN | AMI | GEN | TET |
| *Bacillus* sp. | X | | | Susceptible to all ATM | 0.015 | 1 | 0,25 | 0,25 | 0,25 | 0,12 |
| *Bacillus* sp. | X | | | Susceptible to all ATM | 0.03 | 1 | 0,25 | 0,25 | 2 | 0,12 |
| *Bacillus* sp. | X | | | Susceptible to all ATM | 0.12 | 1 | 1 | 0,5 | 0,5 | 0,5 |
| *Bacillus* sp. | X | | | Susceptible to all ATM | 0.25 | 1 | 128 | 8 | 0,12 | 64 |
| *Bacillus* sp. | X | | | Susceptible to all ATM | 0.25 | 2 | 1 | 1 | 0,5 | 0,12 |
| *Bacillus* sp. | X | | | Susceptible to all ATM | 0.06 | 1 | 0,12 | 0,25 | 1 | 0,25 |
| *Bacillus* sp. | X | | | Susceptible to all ATM | 0.015 | 1 | 0,5 | 0,25 | 1 | 0,12 |
| *Bacillus* sp. | X | | | Susceptible to all ATM | 0.06 | 1 | 0,25 | 0,25 | 1 | 0,5 |
| *Bacillus* sp. | X | | | Susceptible to all ATM | 0.03 | 1 | 1 | 0,25 | 0,12 | 0,12 |
| *Bacillus* sp. | X | | | Susceptible to all ATM | 0.25 | 1 | 0,5 | 0,5 | 0,5 | 2 |
| *Bacillus* sp. | X | | | Susceptible to all ATM | 0.06 | 1 | 0,5 | 0,5 | 0,5 | 0,25 |
| *Bacillus* sp. | X | | | Susceptible to all ATM | 0.03 | 1 | 1 | 0,5 | 1 | 0,12 |
| *Bacillus* sp. | X | | | Susceptible to all ATM | 0.015 | 2 | 0,5 | 0,015 | 0,06 | 0,015 |
| *Bacillus* sp. | X | | | Susceptible to all ATM | 0,5 | 1 | 1 | 0,5 | 0,12 | 0,5 |
| *Bacillus* sp. | X | | | Susceptible to all ATM | 1 | 4 | 0,5 | 0,25 | 0,12 | 0,5 |
| *Bacillus subtilis* | X | | | Susceptible to all ATM | 0,015 | 1 | 0,5 | 0,25 | 0,06 | 0,12 |
| *Bacillus subtilis* | X | | | Susceptible to all ATM | 0,015 | 4 | 2 | 0,5 | 1 | 8 |
| *Bacillus subtilis* | X | | | Susceptible to all ATM | 0,5 | 1 | 0,5 | 0,25 | 0,12 | 0,12 |
| *Bacillus subtilis* | X | | | Susceptible to all ATM | 0.06 | 1 | 0,25 | 0,25 | 0,12 | 0,12 |
| *Bacillus subtilis* | X | | | Susceptible to all ATM | 0.25 | 1 | 16 | 0,25 | 0,12 | 1 |
| *Bacillus subtilis* | X | | | Susceptible to all ATM | 0.25 | 1 | 2 | 0,12 | <0,06 | 0,06 |

1ST, isolates obtained from skin samples previously submitted to the first cycle of treatment with penicillin and streptomycin in the skin bank; *2ND* ,isolates obtained from skin samples previously submitted to the second cycle of treatment with vancomycin; *AMI*, amikacin; *ATM*, antimicrobials; *AZM*, azithromycin; *CFO*, cefoxitin; *CIP*, ciprofloxacin; *CNS*, coagulase-negative staphylococci; *EST*, streptomycin; *GEN*, gentamicin; *MIC*, minimal inhibitory concentration; *PEN*, penicillin; *TET*, tetracycline; *VAN*, vancomycin; *WT*, without treatment.

Table S1. Continued.

| Isolates | Treatment | | | Antimicrobial resistance profile | MIC values (µg/mL) | | | | | |
|---|---|---|---|---|---|---|---|---|---|---|
| | WT | 1ST | 1ST/ 2ND | | PEN | EST | VAN | AMI | GEN | TET |
| *Bacillus subtilis* | X | | | Susceptible to all ATM | 0,03 | 32 | 4 | 0,5 | 0,5 | 16 |
| *Bacillus subtilis* | X | | | Susceptible to all ATM | 0,03 | 32 | 0,5 | 0,12 | <0,06 | 4 |
| *Bacillus subtilis* | X | | | Susceptible to all ATM | 0,03 | 32 | 0,5 | 0,12 | 0,06 | 4 |
| *Bacillus subtilis* | X | | | Susceptible to all ATM | 0,015 | 4 | 0,5 | 0,12 | 0,06 | 4 |
| *Bacillus subtilis* | X | | | Susceptible to all ATM | 0,12 | 16 | 0,5 | 0,5 | 0,12 | 4 |
| *Bacillus subtilis* | X | | | Susceptible to all ATM | 0,06 | 8 | 2 | 0,5 | 0,25 | 16 |
| *Bacillus subtilis* | X | | | Susceptible to all ATM | 0,25 | 2 | 1 | 0,25 | 0,12 | 4 |
| *Bacillus subtilis* | X | | | Susceptible to all ATM | 0,25 | 32 | 0,5 | 0,5 | 0,12 | 8 |
| *Bacillus cereus* | X | | | Susceptible to all ATM | >32 | 2 | 8 | 1 | 2 | 16 |
| *Bacillus cereus* | X | | | Susceptible to all ATM | 8 | 1 | 2 | 0,5 | 1 | 4 |
| *Bacillus vallismortis* | X | | | Susceptible to all ATM | 0.015 | 1 | 0,5 | 0,5 | 0,25 | 0,12 |
| *Bacillus vallismortis* | X | | | Susceptible to all ATM | 0.015 | 1 | 1 | 0,25 | <0,06 | 0,06 |
| *Bacillus pumilus* | X | | | Susceptible to all ATM | 0.03 | 2 | 2 | 0,25 | 1 | 2 |

1ST, isolates obtained from skin samples previously submitted to the first cycle of treatment with penicillin and streptomycin in the skin bank; *2ND* ,isolates obtained from skin samples previously submitted to the second cycle of treatment with vancomycin; *AMI*, amikacin; *ATM*, antimicrobials; *AZM*, azithromycin; *CFO*, cefoxitin; *CIP,* ciprofloxacin; *CNS*, coagulase-negative staphylococci; *EST*, streptomycin; *GEN*, gentamicin*; MIC*, minimal inhibitory concentration; *PEN*, penicillin; *TET*, tetracycline; *VAN*, vancomycin*; WT*, without treatment.

Table S2. Susceptibility profile and antimicrobial MIC values of Gram-positive cocci isolated from human skin allografts with and without antimicrobial treatment.

| **Isolates** | **Treatment** | | | **Antimicrobial resistance profile** | **MIC values (µg/mL)** | | | | | |
|---|---|---|---|---|---|---|---|---|---|---|
| | **WT** | **1ST** | **1ST/ 2ND** | | **PEN** | **EST** | **VAN** | **AMI** | **GEN** | **TET** |
| *Staphylococcus aureus* | X | | | PEN | >32 | 8 | 2 | 1 | 1 | 1 |
| *Staphylococcus aureus* | X | | | PEN | >32 | 8 | 2 | 2 | 1 | 0,5 |
| *Staphylococcus aureus* | X | | | PEN | >32 | 2 | 2 | 1 | 0,25 | 0,5 |
| *Staphylococcus aureus* | X | | | PEN | >32 | 8 | 2 | 0,25 | 1 | 0,25 |
| *Staphylococcus aureus* | X | | | PEN | >32 | 8 | 2 | 1 | 2 | 0,5 |
| *Staphylococcus aureus* | X | | | PEN | >32 | 8 | 2 | 1 | 0,06 | 0,12 |
| *Staphylococcus aureus* | X | | | PEN | 4 | 4 | 2 | 2 | 1 | 1 |
| *Staphylococcus aureus* | X | | | PEN | >32 | 4 | 2 | 2 | 1 | 0,25 |
| *Staphylococcus aureus* | X | | | PEN | 2 | 4 | 2 | 1 | 0,25 | 0,5 |
| *Staphylococcus aureus* | X | | | PEN | >32 | 8 | 2 | 2 | 2 | 0,5 |
| *Staphylococcus aureus* | X | | | PEN | >32 | 16 | 2 | 1 | 2 | 0,5 |
| *Staphylococcus lugdunensis* | X | | | PEN | 0,12 | 4 | 2 | 1 | 1 | 1 |
| Coagulase-negative staphylococci | | X | | CFO | 0,06 | 4 | 4 | 0,5 | 0,25 | 1 |
| Coagulase-negative staphylococci | | X | | AZM | 0,12 | 4 | 2 | 0,25 | 0,25 | 1 |
| *Staphylococcus haemolyticus* | X | | | AZM | 0,03 | 2 | 4 | 0,5 | 0,5 | 0,25 |
| *Staphylococcus haemolyticus* | X | | | AZM | 0,03 | 2 | 2 | 1 | <0,06 | 0,12 |
| *Staphylococcus haemolyticus* | X | | | AZM | 0,06 | 1 | 2 | 0,25 | <0,06 | 0,06 |
| *Staphylococcus haemolyticus* | X | | | AZM | 0,25 | 1 | 2 | 0,5 | 0,25 | 0,12 |
| *Staphylococcus haemolyticus* | X | | | AZM | 0,06 | 2 | 2 | 0,25 | <0,06 | 0,06 |
| *Staphylococcus haemolyticus* | X | | | AZM | 0,06 | 2 | 2 | 0,5 | 0,12 | 0,25 |
| *Staphylococcus haemolyticus* | X | | | AZM | 0,06 | 4 | 2 | 0,25 | 0,25 | 0,5 |

1ST, isolates obtained from skin samples previously submitted to the first cycle of treatment with penicillin and streptomycin in the skin bank; *2ND* ,isolates obtained from skin samples previously submitted to the second cycle of treatment with vancomycin; *AMI*, amikacin; *ATM*, antimicrobials; *AZM*, azithromycin; *CFO*, cefoxitin; *CIP*, ciprofloxacin; *CNS*, coagulase-negative staphylococci; *EST*, streptomycin; *GEN*, gentamicin*; MIC*, minimal inhibitory concentration; *PEN*, penicillin; *TET*, tetracycline; *VAN*, vancomycin*; WT*, without treatment.

Table S2. Continued

| Isolates | Treatment | | | Antimicrobial resistance profile | MIC values (µg/mL) | | | | | |
|---|---|---|---|---|---|---|---|---|---|---|
| | WT | 1ST | 1ST/ 2ND | | PEN | EST | VAN | AMI | GEN | TET |
| *Staphylococcus haemolyticus* | X | | | AZM | 0,12 | 1 | 4 | 0,12 | <0,06 | 0,12 |
| *Staphylococcus haemolyticus* | X | | | AZM | 0,12 | 2 | 2 | 0,12 | <0,06 | 0,12 |
| *Staphylococcus haemolyticus* | X | | | AZM | 0,12 | 1 | 2 | 0,12 | <0,06 | 0,12 |
| *Staphylococcus aureus* | X | | | PEN-CIP | 2 | 4 | 1 | 1 | 1 | 0,25 |
| *Staphylococcus aureus* | X | | | PEN-CIP | >32 | 16 | 2 | 2 | 2 | 0,5 |
| *Staphylococcus aureus* | X | | | PEN-CIP | 4 | 4 | 1 | 1 | 0,25 | 0,25 |
| *Staphylococcus aureus* | X | | | PEN-CIP | >32 | 8 | 2 | 2 | 2 | 0,5 |
| *Staphylococcus aureus* | X | | | PEN-CIP | >32 | 8 | 2 | 0,5 | 0,25 | 0,25 |
| *Staphylococcus aureus* | X | | | PEN-CIP | 0,12 | 8 | 0,25 | 0,12 | <0,06 | 0,12 |
| *Staphylococcus aureus* | X | | | PEN-CIP | 8 | 4 | 2 | 2 | 0,5 | 1 |
| *Staphylococcus epidermidis* | X | | | PEN-CIP | >32 | 16 | 2 | 2 | 2 | 1 |
| *Staphylococcus epidermidis* | X | | | PEN-CIP | >32 | 8 | 4 | 1 | 2 | 1 |
| *Staphylococcus epidermidis* | X | | | PEN-CIP | 2 | 16 | 2 | 1 | 2 | 1 |
| *Staphylococcus epidermidis* | X | | | PEN-AZM | 0,12 | 2 | 2 | 0,5 | 0,5 | 1 |
| *Staphylococcus epidermidis* | X | | | CIP-AZM | >32 | 8 | 2 | 2 | 0,25 | 4 |
| *Staphylococcus epidermidis* | X | | | CFO-CIP-AZM | 2 | 2 | 4 | 2 | 0,5 | 2 |
| *Staphylococcus epidermidis* | X | | | CFO-CIP-AZM | >32 | 4 | 2 | 2 | 2 | 2 |
| *Staphylococcus epidermidis* | X | | | CFO-CIP-AZM | >32 | 4 | 2 | 1 | 0,5 | 1 |
| *Staphylococcus epidermidis* | X | | | CFO-CIP-AZM | >32 | 2 | 2 | 2 | 0,5 | 2 |
| *Staphylococcus epidermidis* | X | | | CFO-CIP-AZM | 8 | 8 | 2 | 2 | 1 | 2 |
| *Staphylococcus saprophyticus* | X | | | CFO-CIP-AZM | 2 | 2 | 2 | 2 | 0,5 | 1 |

1ST, isolates obtained from skin samples previously submitted to the first cycle of treatment with penicillin and streptomycin in the skin bank; *2ND* ,isolates obtained from skin samples previously submitted to the second cycle of treatment with vancomycin; *AMI*, amikacin; *ATM*, antimicrobials; *AZM*, azithromycin; *CFO*, cefoxitin; *CIP*, ciprofloxacin; *CNS*, coagulase-negative staphylococci; *EST*, streptomycin; *GEN*, gentamicin; *MIC*, minimal inhibitory concentration; *PEN*, penicillin; *TET*, tetracycline; *VAN*, vancomycin; *WT*, without treatment.

Table S2. Continued

| Isolates | Treatment | | | Antimicrobial resistance profile | MIC values (µg/mL) | | | | | |
|---|---|---|---|---|---|---|---|---|---|---|
| | WT | 1ST | 1ST/ 2ND | | PEN | EST | VAN | AMI | GEN | TET |
| *Staphylococcus epidermidis* | X | | | PEN-CFO-CIP-AZM | 8 | 8 | 4 | 2 | 0,5 | 2 |
| *Staphylococcus epidermidis* | X | | | PEN-CFO-CIP-AZM | 8 | 8 | 2 | 2 | 0,5 | 2 |
| *Staphylococcus epidermidis* | X | | | PEN-CFO-CIP-AZM | >32 | 8 | 2 | 2 | 1 | 2 |
| *Staphylococcus epidermidis* | X | | | PEN-CFO-CIP-AZM | >32 | 4 | 4 | 1 | 1 | 2 |
| *Staphylococcus epidermidis* | X | | | PEN-CFO-CIP-AZM | 8 | 4 | 2 | 1 | 0,5 | 2 |
| *Staphylococcus epidermidis* | X | | | PEN-CFO-CIP-AZM | >32 | 8 | 2 | 0,5 | 0,5 | 2 |
| *Staphylococcus epidermidis* | X | | | PEN-CFO-CIP-AZM | 1 | 2 | 2 | 1 | 0,25 | 0,5 |
| *Staphylococcus epidermidis* | X | | | PEN-CFO-CIP-AZM | >32 | 2 | 2 | 2 | 4 | 2 |
| *Staphylococcus epidermidis* | X | | | PEN-CFO-CIP-AZM | 8 | 2 | 2 | 2 | 0,5 | 4 |
| *Staphylococcus epidermidis* | X | | | PEN-CFO-CIP-AZM | 16 | 2 | 2 | 2 | 0,5 | 2 |
| *Staphylococcus epidermidis* | X | | | PEN-CFO-CIP-AZM | 2 | 2 | 2 | 1 | 0,5 | 1 |
| *Staphylococcus epidermidis* | X | | | PEN-CFO-CIP-AZM | 8 | 2 | 2 | 2 | 0,5 | 2 |
| *Staphylococcus epidermidis* | X | | | PEN-CFO-CIP-AZM | 16 | 2 | 2 | 2 | 0,5 | 2 |
| *Staphylococcus epidermidis* | X | | | PEN-CFO-CIP-AZM | 8 | 4 | 2 | 0,25 | 0,12 | 0,5 |
| *Staphylococcus epidermidis* | X | | | PEN-CFO-CIP-AZM | 8 | 2 | 2 | 2 | 0,5 | 2 |
| *Staphylococcus epidermidis* | X | | | PEN-CFO-CIP-AZM | >32 | 2 | 2 | 4 | 0,5 | 4 |
| *Staphylococcus epidermidis* | X | | | PEN-CFO-CIP-AZM | >32 | 2 | 2 | 2 | 0,5 | 2 |
| *Staphylococcus epidermidis* | X | | | PEN-CFO-CIP-AZM | >32 | 4 | 2 | 2 | 0,5 | 2 |
| *Staphylococcus epidermidis* | X | | | PEN-CFO-CIP-AZM | 8 | 4 | 4 | 2 | 0,5 | 2 |
| *Staphylococcus epidermidis* | X | | | PEN-CFO-CIP-AZM | 16 | 4 | 2 | 2 | 0,25 | 2 |
| *Staphylococcus epidermidis* | X | | | PEN-CFO-CIP-AZM | 1 | 8 | 2 | 1 | 0,25 | 1 |

1ST, isolates obtained from skin samples previously submitted to the first cycle of treatment with penicillin and streptomycin in the skin bank; *2ND* ,isolates obtained from skin samples previously submitted to the second cycle of treatment with vancomycin; *AMI*, amikacin; *ATM*, antimicrobials; *AZM*, azithromycin; *CFO*, cefoxitin; *CIP*, ciprofloxacin; *CNS*, coagulase-negative staphylococci; *EST*, streptomycin; *GEN*, gentamicin; *MIC*, minimal inhibitory concentration; *PEN*, penicillin; *TET*, tetracycline; *VAN*, vancomycin; *WT*, without treatment.

Table S2. Continued

| Isolates | Treatment | | | Antimicrobial resistance profile | MIC values (µg/mL) | | | | | |
|---|---|---|---|---|---|---|---|---|---|---|
| | WT | 1ST | 1ST/ 2ND | | PEN | EST | VAN | AMI | GEN | TET |
| *Staphylococcus epidermidis* | X | | | PEN-CFO-CIP-AZM | 2 | 4 | 4 | 2 | 0,25 | 2 |
| *Staphylococcus epidermidis* | X | | | PEN-CFO-CIP-AZM | >32 | 4 | 4 | 2 | 0,25 | 0,25 |
| *Staphylococcus epidermidis* | X | | | PEN-CFO-CIP-AZM | 4 | 2 | 2 | 2 | 0,5 | 2 |
| *Staphylococcus epidermidis* | X | | | PEN-CFO-CIP-AZM | 8 | 4 | 2 | 2 | 0,25 | 1 |
| *Staphylococcus epidermidis* | X | | | PEN-CFO-CIP-AZM | 8 | 2 | 2 | 2 | 0,5 | 2 |
| *Staphylococcus epidermidis* | X | | | PEN-CFO-CIP-AZM | >32 | 1 | 2 | 2 | 0,25 | 2 |
| *Staphylococcus epidermidis* | X | | | PEN-CFO-CIP-AZM | >32 | 4 | 4 | 2 | 0,5 | 2 |
| *Staphylococcus epidermidis* | X | | | PEN-CFO-CIP-AZM | 4 | 4 | 2 | 2 | 0,5 | 2 |
| *Staphylococcus epidermidis* | X | | | PEN-CFO-CIP-AZM | 8 | 2 | 2 | 1 | 0,25 | 2 |
| *Staphylococcus epidermidis* | X | | | PEN-CFO-CIP-AZM | 2 | 2 | 2 | 2 | 0,5 | 2 |
| *Staphylococcus epidermidis* | X | | | PEN-CFO-CIP-AZM | 0,5 | 4 | 2 | 1 | 0,12 | 0,5 |
| *Staphylococcus epidermidis* | X | | | PEN-CFO-CIP-AZM | 0,25 | 4 | 2 | 2 | 0,5 | 0,5 |
| *Staphylococcus aureus* | X | | | PEN-CFO-CIP-AZM | >32 | 2 | 2 | 2 | 0,5 | 2 |
| *Staphylococcus saprophyticus* | X | | | PEN-CFO-CIP-AZM | 8 | 2 | 2 | 2 | 0,5 | 2 |
| *Staphylococcus capitis* | X | | | PEN-CFO-CIP-AZM | >32 | 2 | 2 | 8 | 8 | 0,5 |
| *Staphylococcus capitis* | X | | | PEN-CFO-CIP-AZM | >32 | 4 | 2 | 4 | 16 | 1 |
| *Staphylococcus capitis* | X | | | PEN-CFO-CIP-AZM | >32 | 4 | 2 | 2 | 8 | 0,5 |
| *Staphylococcus capitis* | X | | | PEN-CFO-CIP-AZM | >32 | 4 | 2 | 8 | 32 | 0,5 |
| *Staphylococcus capitis* | X | | | PEN-CFO-CIP-AZM | >32 | 4 | 2 | 4 | 16 | 1 |
| *Staphylococcus capitis* | X | | | PEN-CFO-CIP-AZM | >32 | 4 | 2 | 4 | 1 | 0,25 |
| *Staphylococcus capitis* | X | | | PEN-CFO-CIP-AZM | >32 | 2 | 2 | 4 | 8 | 0,5 |

1ST, isolates obtained from skin samples previously submitted to the first cycle of treatment with penicillin and streptomycin in the skin bank; *2ND* ,isolates obtained from skin samples previously submitted to the second cycle of treatment with vancomycin; *AMI*, amikacin; *ATM*, antimicrobials; *AZM*, azithromycin; *CFO*, cefoxitin; *CIP*, ciprofloxacin; *CNS*, coagulase-negative staphylococci; *EST*, streptomycin; *GEN*, gentamicin; *MIC*, minimal inhibitory concentration; *PEN*, penicillin; *TET*, tetracycline; *VAN*, vancomycin; *WT*, without treatment.

Table S2. Continued

| Isolates | Treatment | | | Antimicrobial resistance profile | MIC values (µg/mL) | | | | | |
|---|---|---|---|---|---|---|---|---|---|---|
| | **WT** | **1ST** | **1ST/ 2ND** | | **PEN** | **EST** | **VAN** | **AMI** | **GEN** | **TET** |
| *Staphylococcus capitis* | X | | | PEN-CFO-CIP-AZM | >32 | 2 | 4 | 4 | 8 | 0,5 |
| *Staphylococcus capitis* | X | | | PEN-CFO-CIP-AZM | >32 | 2 | 2 | 4 | 4 | 0,5 |
| *Staphylococcus capitis* | X | | | PEN-CFO-CIP-AZM | >32 | 2 | 2 | 4 | 4 | 0,25 |
| *Staphylococcus capitis* | X | | | PEN-CFO-CIP-AZM | >32 | 2 | 4 | 8 | 16 | 2 |
| *Staphylococcus capitis* | X | | | PEN-CFO-CIP-AZM | >32 | 4 | 2 | 8 | 4 | 0,25 |
| *Staphylococcus haemolyticus* | X | | | Susceptible to all ATM | 0,06 | 2 | 2 | 0,5 | 0,25 | 0,5 |
| *Staphylococcus haemolyticus* | X | | | Susceptible to all ATM | 0,03 | 1 | 2 | 0,25 | <0,06 | 0,06 |
| *Staphylococcus haemolyticus* | X | | | Susceptible to all ATM | 0,03 | 1 | 4 | 0,5 | 0,12 | 0,5 |
| *Staphylococcus lugdunensis* | X | | | Susceptible to all ATM | 0,06 | 4 | 2 | 1 | 0,25 | 0,5 |
| *Staphylococcus lugdunensis* | X | | | Susceptible to all ATM | 2 | 4 | 1 | 0,25 | 0,12 | 2 |
| *Staphylococcus lugdunensis* | X | | | Susceptible to all ATM | 0,03 | 2 | 2 | 0,5 | <0,06 | 0,25 |
| *Staphylococcus lugdunensis* | X | | | Susceptible to all ATM | 0,03 | 4 | 2 | 0,5 | 0,5 | 0,5 |
| *Staphylococcus lugdunensis* | X | | | Susceptible to all ATM | 0,06 | 16 | 2 | 0,5 | <0,06 | 0,25 |
| *Staphylococcus lugdunensis* | X | | | Susceptible to all ATM | 0,015 | 4 | 2 | 4 | 2 | 0,25 |
| *Staphylococcus lugdunensis* | X | | | Susceptible to all ATM | 0,015 | 2 | 2 | 2 | 1 | 1 |
| *Staphylococcus lugdunensis* | X | | | Susceptible to all ATM | 0,06 | 4 | 2 | 1 | 1 | 0,5 |
| *Staphylococcus lugdunensis* | X | | | Susceptible to all ATM | 0,06 | 4 | 1 | 1 | 1 | 1 |
| *Staphylococcus lugdunensis* | X | | | Susceptible to all ATM | 0,06 | 2 | 2 | 1 | 1 | 1 |
| *Staphylococcus lugdunensis* | X | | | Susceptible to all ATM | 0,06 | 4 | 2 | 0,25 | 0,06 | 0,12 |
| *Staphylococcus lugdunensis* | X | | | Susceptible to all ATM | 0,06 | 4 | 2 | 0,5 | 0,5 | 0,5 |

1ST, isolates obtained from skin samples previously submitted to the first cycle of treatment with penicillin and streptomycin in the skin bank; *2ND* ,isolates obtained from skin samples previously submitted to the second cycle of treatment with vancomycin; *AMI*, amikacin; *ATM*, antimicrobials; *AZM*, azithromycin; *CFO*, cefoxitin; *CIP*, ciprofloxacin; *CNS*, coagulase-negative staphylococci; *EST*, streptomycin; *GEN*, gentamicin; *MIC*, minimal inhibitory concentration; *PEN*, penicillin; *TET*, tetracycline; *VAN*, vancomycin; *WT*, without treatment.
